# The Effect of the Long-Term Calcipotriol/Betamethasone Dipropionate Local Therapy on Tissue Resident Memory Cells Markers in Psoriatic Eruptions

**DOI:** 10.3390/ijerph19148345

**Published:** 2022-07-08

**Authors:** Marta Kasprowicz-Furmańczyk, Joanna Czerwińska, Waldemar Placek, Agnieszka Owczarczyk-Saczonek

**Affiliations:** Department of Dermatology, Sexually Transmitted Diseases and Clinical Immunology, University of Warmia and Mazury in Olsztyn, 10-719 Olsztyn, Poland; joannaj061@gmail.com (J.C.); w.placek@wp.pl (W.P.); aganek@wp.pl (A.O.-S.)

**Keywords:** psoriasis, tissue resident memory cells, TRM, immune memory, calcipotriol, betamethasone dipropionate

## Abstract

Background: The natural course of psoriasis is characterized by the long-term persistence of lesions and a predilection for relapse in the same area. It is caused by the inherence of TRM (tissue resident memory T cells) in apparently healthy skin. These cells are able to initiate an inflammatory cascade and induce relapse of the disease. These cells are characterized by high resistance to damaging factors and apoptosis, which determines their longevity. Aim: The aim of our study was to evaluate the presence of TRM in psoriatic plaques before, during and after 12 weeks of therapy in patients treated with topical calcipotriol and betamethasone dipropionate (Cal/BD) foam. Methods: TRM markers (CD4, CD8, CD103, CD69, CD49, CXCR6) and tissue expression of cytokines (IL-17A, IL-22) in the lesional psoriatic skin from 10 patients compared to 10 healthy skin samples were estimated by immunohistochemistry. Biopsy samples from the area of the same psoriatic plaque were collected three times: before the initiation of therapy, 4 and 12 weeks after its initiation. Results: The presence of TRM markers in the epidermis and dermis of psoriatic lesions was significantly higher when compared to the skin of control group patients. A reduction in the expression of the characteristic TRM markers (CD8, CD4, CD103, CD69, CXCR6, IL-17A and IL-22) was observed in the epidermis on week 12 of therapy, while a depletion in the expression of TRM in the dermis was demonstrated only in CD4 and IL-22. Conclusions: Topical treatment with Cal/BD foam significantly decreased the expression of TRM markers mainly in the epidermis, and to a lesser extent in the dermis, during the 12-week observation period. It probably results from a worse penetration of the drug into the dermis and the effect of the preparation mainly on the epidermis. The persistence of a high expression of TRM markers in the dermis may result in the rapid recurrence of lesions after discontinuation of topical treatment.

## 1. Introduction

There were 4,622,594 (95% uncertainty interval) incident cases of psoriasis worldwide in 2019 [1]. Psoriasis is a chronic and recurrent inflammatory skin disease mediated by T lymphocytes. It is characterized by the appearance of skin lesions in certain places and relapses of the disease in the same location [2]. The recurrent nature of psoriasis is related to the immune memory in the skin and likely depends on tissue resident long-lived memory T cells (TRM) [3]. These cells are able to initiate the inflammatory cascade and thus contribute to disease relapse in apparently healthy skin [4]. Both CD4+ and CD8+ TRM have been described not only in the skin but also in other tissues, including the mucosa, brain, gastrointestinal tract, lungs and pancreas [3]. Resident memory T cells remain in peripheral tissues for extended periods of time, are non-recirculating and assure the first line of adaptive immune responses in resident tissues [5,6]. TRM differ physiologically from circulating T cells in their expression of tissue residence markers such as CD69 and CD103 and in their distinguishing profile of transcription factors [7]. There are two main types of TRM: CD8+, which predominates in the psoriatic epidermis and express mainly the CD103, CD69, CD49a, and TRM CD4+, which are located close to the blood vessels in the dermis and have a high proliferative capacity [8]. The remaining molecules that differentiate TRM from other types of circulating memory T cells are CD101, CXCR3 and CXCR6 [4,8]. CD8+ TRM in the skin of patients suffering from psoriasis express the IL23 receptor, and, therefore, are able to produce pro-inflammatory interleukin (IL)-17 and IL-22 in the psoriatic skin, even for many months after remission [4,9]. In psoriasis, CD8+ TRM-producing IL-17A are considered to be one of the pathogenic skin populations involved in the pathogenesis of the disease [10].

Although TRM and their role in autoinflammatory diseases have been the subject of research for several years, we still do not have many reports on the effect of specific therapies on the presence of TRM in the skin of patients who are treated general and topical for plaque psoriasis. It is known that these cells are highly resistant to damaging factors and apoptosis, which probably determines their longevity. A significant positive dependence has been demonstrated between the expression of TRM markers in patients with psoriasis vulgaris and the persistence of skin lesions [8,11]. The rapid relapse of psoriatic lesions in the same localization after the action of the trigger factor may be explained by the longevity and accumulation of TRM in the skin of psoriatic patients [4].

## 2. Methods

### 2.1. Study Group

The study group included 10 patients of the Outpatient Clinic at the Department of Dermatology, Sexually Transmitted Diseases, and Clinical Immunology in Olsztyn with plaque psoriasis, without psoriatic arthritis, untreated for at least 4 weeks. The inclusion criterion for the study group was a Psoriasis Area Severity Index (PASI) lower than 10 and a body surface area (BSA) lower than 10%. Patients suffering from chronic and acute inflammatory diseases and dermatoses (other than psoriasis), neoplastic diseases, heart, kidney and liver failure, and nicotine or alcohol addiction were excluded. The control group included healthy volunteers (*n* = 10) with no personal or family history of psoriasis and no concomitant autoimmune and inflammatory disorders. Skin samples from psoriasis patients were obtained from psoriatic plaques which were relapsing at the same sites after the end of previous treatment three times—before the initiation of therapy (week 0), after 4 and 12 weeks of treatment. Patients used the topical drug containing Cal/BD once a day for the 4 subsequent weeks, then as a maintenance (proactively) twice a week. Healthy volunteer skin samples were collected from surgical wastes left after removal of pigmentary lesions located on the trunk or limbs. In the study group, we also assessed the severity of the disease using the following scales: PASI, BSA, DLQI.

### 2.2. Clinical Samples

Assessment of TRM in biopsy specimens was performed by the immunofluorescence method according to those described previously [8], with modifications. We obtained three 4 mm punch biopsies per patient from lesional skin and one from healthy volunteers (non-lesional skin), using local anesthesia (1% lignocaine). Tissue samples were cut into 5 μm thick sections in the cryostat (SLEE MEV, Nieder-Olm, Germany) and mounted onto glass slides coated with poly-L-lysine (Menzel-Glaser, Braunschweig, Germany). Sections were fixed in acetone for 10 min and rinsed in 0.01 M PBS. Next, they were incubated with 2.5% normal horse serum for 30 min at room temperature (Vector Laboratories, Burlingame, CA, USA) to decrease nonspecific binding. Sections were incubated at 4 °C overnight with mouse anti-CD8 and CD4 or rabbit anti-CD103, CD69, CD49, CXCR6, IL-17A and IL22 polyclonal antibody (1:50; Merck Millipore, Billerica, MA, USA). On the following day, the sections were washed in PBS and incubated for 30 min with secondary horse anti-mouse/rabbit antibodies (commercially diluted; ImmPRESS Universal reagent Anti-Mouse/Rabbit Ig; Vector Laboratories, Burlingame, CA, USA). In negative controls, primary and/or secondary antibodies were replaced with 0.01 M PBS. The immunohistochemical specimens were evaluated under a fluorescent microscope (CH30/CH40; Olympus, Tokyo, Japan).

The used analysis of images was based on those previously described by the Leska et al. method [12]. TRM staining was performed in triplicates (three slides for each visit of every patient). Next, images of five different areas for epidermis and dermis were taken (under 500 magnification), resulting in 15 images per one visit (separately for epidermis and dermis). The same procedure was performed in for the healthy control. The images were analyzed semiquantitatively. According to the previous publications as the assessed parameter, the immunoreactive area (the ratio of the area occupied by the immunopositive cells to the total area occupied by epidermis/dermis) was chosen. The level of each TRM immunoreactivity was measured using ImageJ using a threshold function to select a range of grey values that were optically identified as positive staining. In the statistical analysis, percentage data were transformed arcsin.

The results were assessed statistically by a non-parametric Mann–Whitney U test. The results are expressed as means ± SEM. Correlation between proteins was analyzed by Spearman’s test (*p* < 0.05). All calculations were performed using the Statistica software, release 13 (Statsoft, Inc., Tulsa, OK, USA). Values *p* < 0.05 were considered as statistically significant.

The study was approved by the Bioethical Committee of the Warmia and Mazury University in Olsztyn (Resolution 24/2020). Informed consent was obtained from each patient enrolled in the study.

## 3. Results

### 3.1. Immunoreactive Area and Skin Localization of TRM Markers in Lesional Skin before Treatment in Comparison with Healthy Control (Week 0)

**Figure 1:**
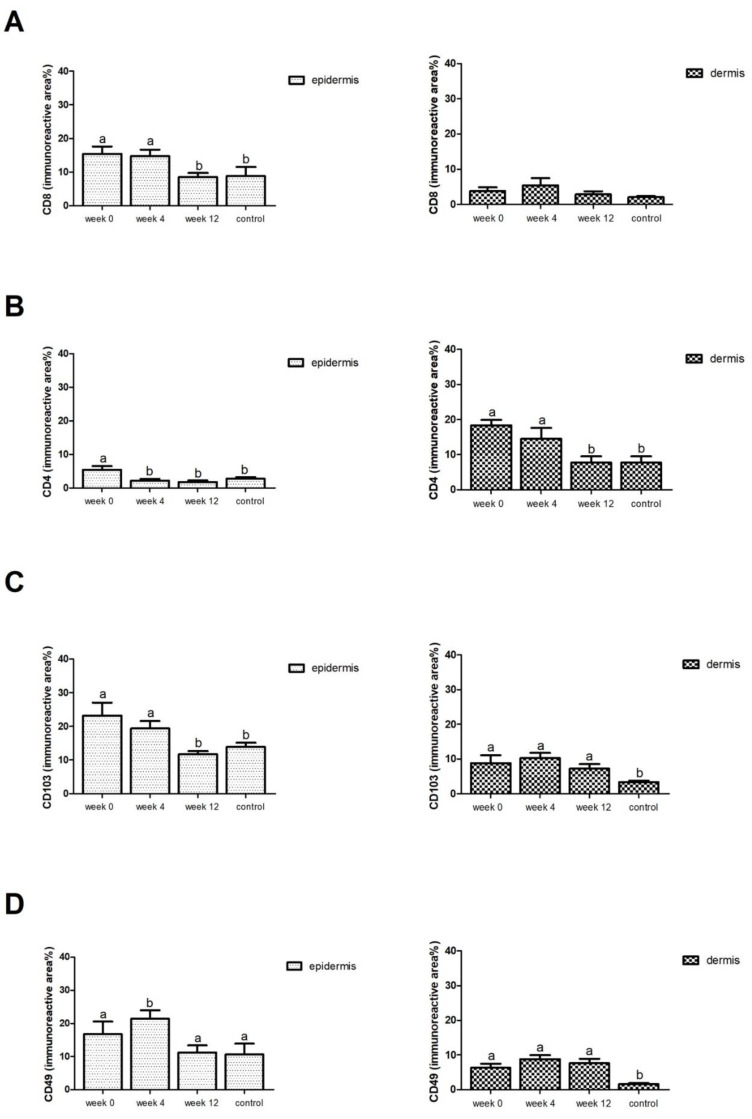

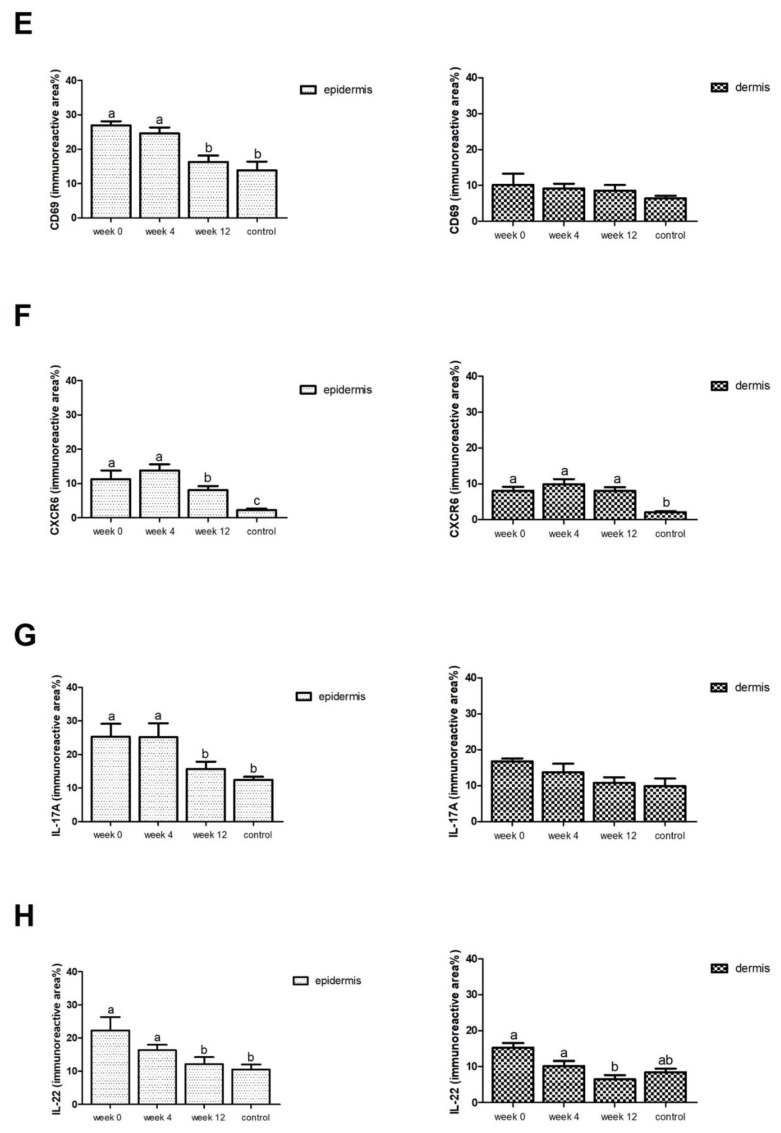
Immunoreactive area (means ± SEM) of TRM (in epidermis and dermis separately) of (**A**) CD8, (**B**) CD4, (**C**) CD103, (**D**) CD49, (**E**) CD69, (**F**) CXCR6, (**G**) IL-17A, (**H**) IL-22 in lesional skin during week 0, 4 and 12 (*n* = 10) in comparison with healthy control (*n* = 10, dermis and epidermis). Different letters indicate significant differences (*p* < 0.05) between the various measurement points, whereas the same letters indicate a lack of differences (*p* < 0.05).

In patients with psoriasis, an increase in the CD8 and CD69, IL-17A and IL-22 immunoreactive area in the epidermis was found, but it did not change significantly in the dermis compared to the control group. In the case of CD103, CXCR6, an increase in the immunoreactive area in both the epidermis and the dermis was found compared to the control group. This was similar in the case of the CD4 immunoreactive surface, where the amount of the marker was dominant in the dermis and not in the epidermis, as in the case of CXCR6 (Figure 1).

### 3.2. Immunoreactive Area and Skin Localization of TRM Markers in Lesional Skin after 4 Weeks of Treatment (Week 4)

After 4 weeks of therapy, a statistically significant decrease in the immunoreactive area was observed only in the case of CD4 markers in the epidermis. Other markers were also reduced, but no statistically significant changes were achieved in the epidermis and dermis (Figure 1).

### 3.3. Immunoreactive Area and Skin Localization of TRM Markers in Lesional Skin after 12 Weeks of Treatment (Week 12) in Comparison with Healthy Control

Statistically significant decreases in fluorescence area compared to week 0 were observed for the markers: CD8 (8.51 ± 1.2% vs. 15.43 ± 2.1%); CD4 (1.78 ± 0.4% vs. 5.46 ± 1.1%); CD103 (11.72 ± 0.9% vs. 23.26 ± 3.8%); CD49 (16.79 ± 3.7% vs. 11.23 ± 2.1%); CD69 (16.31 ± 1.8% vs. 26.98 ± 1.1%); CXCR6 (8.10 ± 1.1% vs. 11.21 ± 2.5%), IL-17A (15.71 ± 2.1% vs. 25.27 ± 3.8%) and IL-22 (12.16 ± 2.0% vs. 22.29 ± 3.8%) within the epidermis.

It was completely different in the case of the dermis. We observed a decrease in the immunofluorescent surface only in the case of: CD4; IL-22 (Table 1).

**Figure 2:**
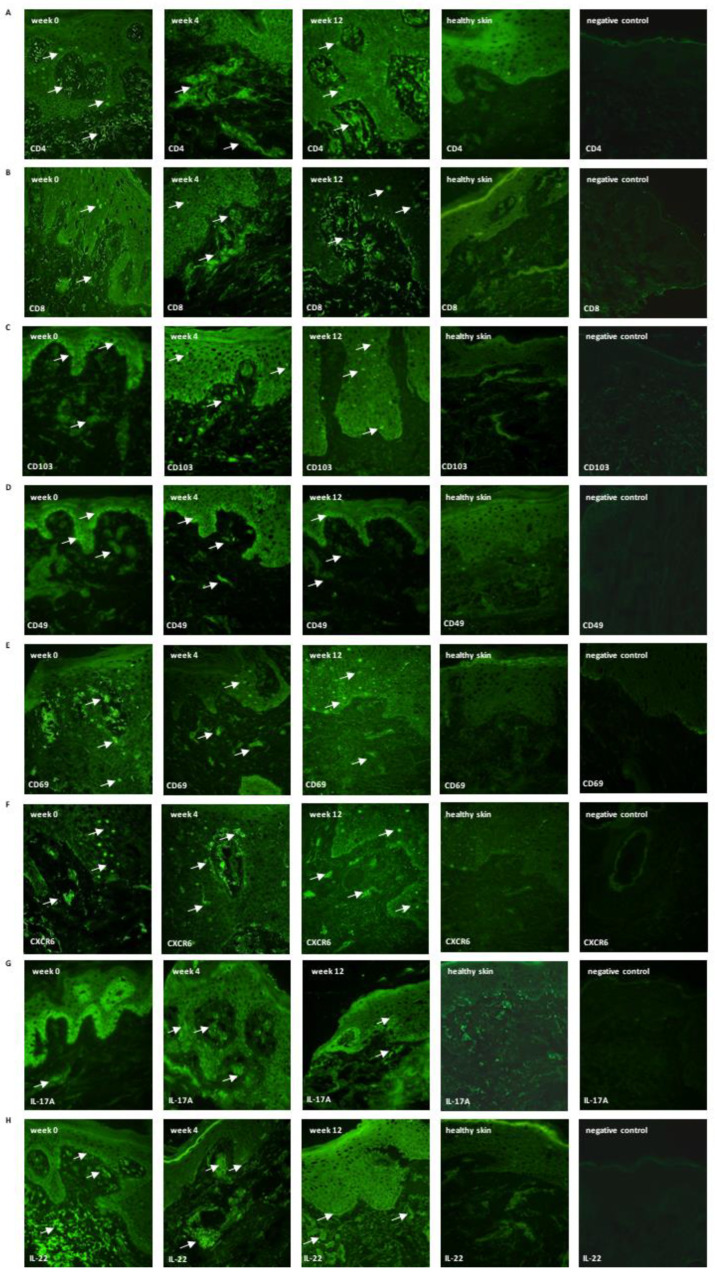
Skin localization of (**A**) CD8, (**B**) CD4, (**C**) CD103, (**D**) CD49, (**E**) CD69, (**F**) CXCR6, (**G**) IL-17A, (**H**) IL-22 (the representative sections): lesional skin during week 0, 4, and 12 (*n* = 10); healthy skin (*n* = 10) and negative control (without primary antibody). The proteins are marked in green (white arrows, fluorescein). Magnification: 500×.

After 12 weeks, the immunofluorescent surface of TRM markers in patients’ epidermis and dermis similar to the control group was observed in the case of: CD8; CD4; CD69; IL17; IL-22 (Figure 1 and Figure 2).

After 12 weeks of treatment in all patients in the study group, we also found a statistically significant clinical improvement in the severity of the disease—as measured by the PASI and BSA scales, and an improvement in quality of life measured by the DLQI (Table 2).

## 4. Discussion

To date, no registered drugs are available that directly and specifically inhibit activity of skin TRM; however, current treatments for psoriasis are known to affect TRM of skin with psoriasis [10,13]. There are still few studies available in the literature describing the effect of systemic therapy—biological drugs, systemic drugs and phototherapy—on TRM in the skin of patients. Even less is known about the effects of various topical treatments on TRM in patients’ skin. While therapies targeting tumor necrosis factor (TNF)-α, IL-23 and IL-17 have been proven effective in psoriasis, topical treatment remains the method of choice in most cases. This is due to the severity of skin lesions and the convenience, safety and economic effectiveness of local therapy. Among the topical medications, the most commonly used are glucocorticosteroid preparations, calcineurin inhibitors and vitamin D3 derivatives (vitamin D3 analogues) [14]. The popular drug of the first choice is a combined preparation containing betamethasone dipropionate at a dose of 0.5 mg/g and calcipotriol at a dose of 50 µg/g. Therefore, we decided to assess the effect of topical therapy with a combined foam preparation containing betamethasone dipropionate and calcipotriol on the number of TRM markers in the area of skin lesions subjected to 12-week therapy.

In psoriasis, the balance between the Th1 and Th2 immune response pathways is disturbed, with a predominance towards Th1 [15]. Activated Th1 cells secrete large amounts of TNF-α and interferon γ (IFN-γ), activating dendritic cells (DCs) and stimulating keratinocytes. On the other hand, activation of Th17 and Tc17 lymphocytes leads to an increased production of cytokines such as IL-17A and IL-22, which enhance excessive proliferation and abnormal differentiation of the epidermis [16,17,18]. In plaque psoriasis, vitamin D and its analogs work mainly by inhibiting proliferation and inducing keratinocyte differentiation by inducing inhibition of transforming growth factor β and cyclin-dependent kinase inhibitors. In addition, vitamin D has an immunomodulatory effect by inhibiting the activation and differentiation of Th17/Th1 cells and inducing a Th2/Threg response [19]. Betamethasone dipropionate belongs to the group of synthetic, fluorinated glucocorticosteroids with a strong effect (class III according to the WHO classification). Glucocorticosteroids in psoriasis suppress the immune system, especially the pro-inflammatory cytokines and chemokines, and thus inhibit the activation of T cells [18]. Betamethasone dipropionate and calcipotriol in the combined preparation complement each other in terms of immunosuppressive, immunomodulating and antiproliferative effects [18]. The secretion of TNF-α by Tc1 and Th1 lymphocytes is inhibited, which prevents the subsequent activation of DCs and keratinocytes, and these substances have an additive effect on reducing IL-17 production by Th and Tc cells [20,21]. The effectiveness of the combination of these drugs is supported by the fact that GCS inhibit both Th1 and Th2 immune mechanisms, while vitamin D3 derivatives have an opposite effect in the Th2 response, which in total results in a slight induction of the Th2 pathway. Balancing the Th1/Th2 ratio may prevent the rebound phenomenon after the end of therapy [18,22].

Dyring et al. investigated the impact of the vitamin D analogue—calcipotriol on the incidence of CD4+ and CD8+ T cells and innate lymphoid cells (ILC) and their production of IL-17A, IFN-γ and IL-22 in psoriatic plaques in patients suffering from psoriasis vulgaris. The authors observed after 14 days of treatment with calcipotriol, a relevant clinical and histological effect; however, they found no significant differences in the incidence of CD4+ and CD8+ T cells or ILC between calcipotriol-treated skin and vehicle. Interestingly, the authors revealed that calcipotriol reduces the incidence of CD8+ IL-17+ T cells in psoriatic plaques, accompanied by clinical and histological improvement. No changes in the frequency of IL-22+ or IFN-γ+ cells were observed [23]. In various studies on the effectiveness of calcipotriol in monotherapy, divergent results were observed for CD4+ and CD8+—some authors reported a significant decrease in these cells after 4 weeks of therapy [24,25], while others found no significant changes in the amount of CD4+ and CD8+ [23,26,27].

Much research has also been done on the combined efficacy of topical corticosteroids and vitamin D3 analogues. Most reports found combination therapy to be more effective than a single agent in terms of therapeutic efficacy or anti-inflammatory response [21]. During short-term therapy Cal/BD, Dyring et al. did not observe any significant changes in the amount of IL-22 [23]. On the other hand, Ikeda et al. showed a reduction in the level of IL-22 [28].

Fujiyama et al. assessed the effect of topical treatment in monotherapy with calcipotriol or betamethasone, and with two-component combination therapy on psoriatic plaques during 14 days of therapy. In all samples, clinical and histological improvement was achieved and a reduction of IL-17A+ cell infiltration occurred, but the best effectiveness was shown by two-component therapy. There was observed clinical, histologic and IL-17A+ cell-infiltrate improvement [21]. Additionally, evaluation of the number of ex vivo expanded T cells showed the greatest decrease by topical application of combination therapy. The number and frequency of Th17 cells were significantly reduced by Cal/BD and Cal, suggesting that Cal has the ability to selectively suppress Th17 cells.

Vissers et al. determined the effect of combining calcipotriol ointment once daily and betamethasone dipropionate ointment once daily compared to the effect of using each drug twice daily alone over a four-week treatment period. Markers of epidermal proliferation (Ki-67) and epidermal differentiation (keratin-10) were assessed, and a quantitative analysis of the image and subsets of T lymphocytes in the epidermis and dermis (CD4, CD8, CD25, CD45RO, CD45RA, CD94, CD161 and CD2) was performed. Calcipotriol was shown to have a large effect on the Ki-67 proliferation marker and the keratin-10 epidermal differentiation marker, while the effect on T cell subsets was more selective with significant reductions in CD45RO+ and CD8+ T cells. In contrast, the effect of betamethasone dipropionate on the epidermis was limited to normalization of differentiation with a highly significant increase in the surface of the epidermis positive for keratin-10 with no significant effect on the Ki-67 positive nuclei, and the effect on the T cell subsets was limited to the depletion of NK T receptors labeled as CD94 and CD161 in the epidermis. The coupling of the two therapies had no effect on the proliferation marker Ki-67 and the keratin-10 marker, over and above the effect of calcipotriol monotherapy. Moreover, the combination of the two approaches reduced virtually all T-cell subsets, and the efficacy was significantly higher than that of monotherapy [24].

When assessing the presence of TRM markers in psoriatic skin lesions before, during and after 12-week therapy in patients treated with topical Cal/BD, we showed that this therapy reduces the expression of TRM markers mainly in the epidermis, but does not significantly influence the expression of these markers in the dermis. Reduced expression was observed with CD8, CD103, CD69, CD4, CXCR6, IL-17A and IL-22 within the epidermis, and in the dermis, only CD103, CD4 and IL-22 decreased in the immunofluorescent surface area (Figure 1 and Figure 2). This is probably due to the lower penetration of topical drugs into the dermis and acting mainly at the epidermis level. Interestingly, in another study we conducted [29] comparing the effect of various systemic therapies (methotrexate, adalimumab, anti-IL-17) on the amount of TRM in different layers of the skin, a decrease in fluorescence was found for each treatment. The fastest response, already in the 4th week of treatment, was observed in the case of anti-IL17, while in the case of methotrexate and adalimumab, a statistically significant decrease was observed in the 12th week of therapy. An interesting conclusion from the study was the fact that in the case of all the above-described therapies, we observed a decrease in immunofluorescence in the dermis, and not in the epidermis, after 12-week follow-up [29]. Similar conclusions were reached by Kurihara et al., observing the resistance of CD8+ TRM in the epidermis to general treatment (CsA, PDE4 and biological) [13]. Taking into account the literature reports and the results we have published, it seems that combining general therapy with local therapy may have an impact on achieving a more effective reduction of TRM in both the dermis and epidermis. Perhaps this would have a potential impact on the duration of remission?

It is not known how long treatment should take to completely eliminate TRM and prevent rapid relapses, possibly depending on the therapy used. Relapses are common, especially with reactive treatments, since TRM remain capable of initiating an inflammatory cascade and inducing relapses of psoriatic plaques despite clinical resolution of the skin lesions. These cells are characterized by a high resistance to damaging factors and apoptosis, and also have the ability to transform into long-lived TRM. A long-term, proactive approach to treatment, including supportive care after successful initial treatment, can help to prolong disease remission and improve clinical outcomes, and may positively impact patients’ quality of life [30]. The results of the Phase III PSO-LONG trial showed that long-term proactive management was superior to reactive management in reducing the number of relapses and extending the days of remission in adult patients with plaque psoriasis [31]. In our study, a statistically significant effect of local treatment Cal/BD measured by a decrease in the immunofluorescent surface area of specific TRM markers was observed only in the 12th week of therapy (CD8, CD103, CXCR6, IL-17A, IL22), and in the 4th week of treatment, only CD69 and CD4 decreased. Perhaps longer periods of remission and a reduction in the number of relapses during proactive therapy are associated with a significant reduction in TRM in the skin of patients. In the case of most topical medications, according to the characteristics of medicinal products, treatment can be continued for max. 3–4 weeks, which, based on our research, seems to be insufficient time to achieve TRM reduction. So far, only Cal/BD can be used in the treatment of psoriasis in proactive therapy (4 weeks of treatment once a day, then maintenance twice a week).

## 5. Conclusions

Cal/BD local therapy reduces the expression of TRM markers mainly in the epidermis but does not significantly affect their expression in the dermis.

The decrease in markers was observed in the 12th week of therapy, which confirms the validity of proactive therapy in the local treatment of plaque psoriasis. Four weeks of continuous therapy did not significantly reduce the amount of TRM in the skin of patients with psoriasis vulgaris.

## 6. Limitations

The small sample size is a limitation of this study. The study required a triple biopsy at the same site, which was difficult for the patient to accept. The studies are still in progress; a longer follow-up period is recommended (repeated biopsy after 24 and 52 weeks), which would enable further evaluation of changes in TRM markers and would also be enable their correlation with the duration of disease remission. Another weakness of the study is related to technical limitations. It would be advisable to use more markers in immunofluorescence and to perform co-staining of the sections to increase the specificity of the method.

## Figures and Tables

**Table 1:** Immunoreactive area (%) of TRM markers (X ± SEM) in lesional skin (*n* = 10, dermis and epidermis) at week 0, week 4 and week 12 in comparison with healthy control (*n* = 10, dermis and epidermis). Asterisks show the statistically significant differences between analyzed groups (week 0 and 12). ↓—means decrease, ↑—means increase.

	Week 0		Week 4		Week 12		Changes from Week 0 to 12 (%)		Healthy Control	
	Epidermis	Dermis	Epidermis	Dermis	Epidermis	Dermis	Epidermis	Dermis	Epidermis	Dermis
**CD8**	15.43 ± 2.1	3.83 ± 1.0	14.74 ± 1.9	5.46 ± 2.1	8.51 ± 1.2	2.84 ± 0.9	↓45 *	↓15	8.83 ± 2.68	2.04 ± 0.33
**CD4**	5.46 ± 1.1	18.34 ± 1.5	2.17 ± 0.5	14.5 ± 3.0	1.78 ± 0.4	7.71 ± 1.8	↓67 *	↓57 *	2.77 ± 0.49	7.71 ± 1.80
**CD103**	23.26 ± 3.8	8.81 ± 2.2	19.38 ± 2.1	10.25 ± 1.5	11.72 ± 0.9	7.23 ± 1.3	↓49 *	↓17	13.88 ± 1.28	3.37 ± 0.42
**CD49**	16.79 ± 3.7	6.31 ± 1.1	21.40 ± 2.5	8.78 ± 1.1	11.23 ± 2.1	7.6 ± 1.2	↓33 *	↑20	10.66 ± 3.23	1.55 ± 0.34
**CD69**	26.98 ± 1.1	10.16 ± 3.0	24.57 ± 1.7	9.1 ± 1.3	16.31 ± 1.8	8.58 ± 1.5	↓39 *	↓15	13.89 ± 2.51	6.39 ± 0.69
**CXCR6**	11.21 ± 2.5	8.02 ± 1.1	13.7 ± 1.8	9.92 ± 1.3	8.10 ± 1.1	8.03 ± 0.9	↓27 *	0	2.24 ± 0.41	2.05 ± 0.33
**IL17**	25.27 ± 3.8	16.76 ± 0.8	25.17 ± 4.1	13.69 ± 2.4	15.71 ± 2.1	10.76 ± 1.5	↓37 *	↓35	12.45 ± 0.94	9.85 ± 2.14
**IL22**	22.29 ± 3.8	15.26 ± 1.3	16.29 ± 1.6	10.22 ± 1.3	12.16 ± 2.0	6.52 ± 1.1	↓45 *	↓57 *	10.54 ± 1.44	8.45 ± 1.03

**Table 2:** Disease activity measured by PASI, BSA and DLQI scales in patients (*n* = 10) treated with calcipotriol/betamethasone dipropionate foam during week 0, 4 and 12. Asterisks show the statistically significant differences between week 0 and week 12. ↓—means decrease, ↑—means increase.

	Week 0	Week 4	Week 12	Changes from Week 0 to 12 (%)	
**PASI**	6.91 ± 0.8	3.32 ± 0.59	2.1 ± 0.34	↓69 *	*p* = 0.0008
**BSA**	8.13 ± 1.09	5.62 ± 0.98	3.12 ± 0.35	↓61 *	*p* = 0.002
**DLQI**	11.18 ± 0.86	8 ± 1.11	6 ± 1.08	↓46 *	*p* = 0.02

## Data Availability

Not applicable.
